# The Use of Ultra-Small Fe_3_O_4_ Magnetic Nanoparticles for Hydrothermal Synthesis of Fe^3+^-Doped Titanate Nanotubes

**DOI:** 10.3390/ma13204612

**Published:** 2020-10-16

**Authors:** Maciej Marć, Lidia Najder-Kozdrowska, Nikos Guskos, Grzegorz Żołnierkiewicz, Ana Maria Montero, Mirosław Roman Dudek

**Affiliations:** 1 Institute of Physics, University of Zielona Góra, ul. Szafrana 4a, 65-069 Zielona Góra, Poland; l.najder-kozdrowska@if.uz.zgora.pl (L.N.-K.); m.dudek@if.uz.zgora.pl (M.R.D.); 2 Institute of Physics, West Pomeranian University of Technology, Al. Piastów 17, 70-310 Szczecin, Poland; nikos.guskos@zut.edu.pl (N.G.); grzegorz.zolnierkiewicz@zut.edu.pl (G.Ż.); 3 Aachen Institute for Advanced Study in Computational Engineering Science, RWTH, 52062 Aachen, Germany; anamtr94@gmail.com

**Keywords:** titanate nanotube, magnetic nanoparticle, magnetic resonance, photocatalysis

## Abstract

A method of the hydrothermal synthesis of Fe^3+^-doped titanate nanotubes (TNT) is reported in which the ultra-small Fe_3_O_4_ nanoparticles are used as the sources of Fe^3+^ ions. The magnetic nanoparticles with a diameter of about 2 nm are added during the washing stage of the hydrothermal procedure. During washing, they gradually degrade and at the same time, the titanate product is transformed into nanotubes. The obtained nanotubes were characterized by structural and magnetic measurements. It was found that, depending on the value of the external magnetic field, they may show the property of room temperature ferromagnetism, paramagnetism or they may be diamagnetic. It was also shown that the modified TNTs have greater photocatalytic activity compared to unmodified TNTs.

## 1. Introduction

Simple and low cost methods of preparing various forms of titanium dioxide (TiO_2_), such as nanoparticles, flakes, or nanotube structures and a wide spectrum of applications for them cause a growing interest in these materials [1,2]. The attractiveness of TiO_2_-based materials increased significantly due to the possibility of using them for the conversion of solar energy to chemical one. Currently, issues related to their use for solar cells [3,4], photocatalysis [5,6,7] or degradation of organic compounds [8] are the subjects of very intense research. It is worth to add that TiO_2_ is widely regarded as not harmful to human health [9]. It represents n-type semiconductor but the certain disadvantage is the presence of a large bandgap moving the edge of light absorption towards the ultraviolet region. The bandgap is 3.0 eV and 3.2 eV for bulk forms of rutile and anatase, respectively, and increases for nanostructures, e.g., it is equal to 3.3 eV for titanate nanotubes [10,11]. In TiO_2_, the lowest energy edge in the conduction band is due to vacant Ti^4+^ bands and the upper energy edge of the valence band is determined by the filled $O_{2}^{-}$ bands [12,13]. Recently, there have appeared numerous publications showing the possibility of decreasing the width of the bandgap by using the appropriate doping procedures [11,13,14,15,16]. In particular, the substitutional Fe^3+^ ions are charge compensated by the oxygen vacancies and move Fermi level, E*_F_*, towards valence band [13,17,18,19]. In [20], it was shown that at a high concentration of Fe^3+^, TiO_2_ may even behave like a p-type semiconductor.

In this work, we focus on Fe^3+^-doping of titanate nanotubes (TNTs). The nanotubes are prepared by an alkaline hydrothermal method [21] which is commonly used next to the anodization method [22] and template method [23]. A wide discussion on the hydrothermal treatment of TiO_2_ nanoparticles can be found in [10,24,25,26]. Transforming TiO_2_ nanopowder into TNTs yields a material with very large specific surface area [27] which, also possesses an ion-exchangeable layered structure [28]. Further modifications of TNTs by ion doping or decorating its surface with organic molecules and nanoparticles can enhance the adsorption properties of TNTs [29,30,31]. An additional advantage is that the titanate nanotubes does not contain toxic substances, as shown by recent studies [32,33].

The methods of doping titanate nanotubes with ions depend on the specific application. The examples of ions that are often used are Fe^3+^ [34,35,36,37,38], Co^2+^ [39,40,41], Ni^2+^ [39], Mn^2+^ [42], Gd^3+^ [43], Yb^3+^ [44], Nd^3+^ [44], Au^3+^ [45], Pt^2+^ [45]. They show how diverse the methods of doping TiO_2_ nanotubes are. It should be noted that ion doping methods can also apply to TiO_2_ material in the form of nanoparticles, e.g., Fe^3+^-doping [46,47,48,49,50]. In the present work, we suggest a method for Fe^3+^-doping titanate nanotubes using ultra-small Fe_3_O_4_ nanoparticles with a diameter of about 2 nm. Such an extremely small size of nanoparticles causes that they quickly degrade in an acidic environment, even at a very low acid concentration. As a consequence, at the stage of the washing procedure, previously added nanoparticles degrade and become sources of high concentration of iron ions located on the surface of nanotubes. This is a novelty of this method compared to others that use ultra-small iron oxide nanoparticles deposited on the surface of titanate nanotubes. An example may be the paper [51], where nanoparticles with an average size of 10 nm were used to coat titanate nanotubes. In this case, the nanoparticles did not degrade and they influenced the diameter of the nanotubes. Our method is also complementary to the methods of delivering iron ions directly from aqueous iron (III) chloride solution as in paper [34] where a microwave-assisted hydrothermal method was used. It is also worth mentioning the method which is a combination of the sol-gel method which produces Fe-doped TiO_2_ powder and the hydrothermal method for the synthesis of nanotubes as in the publication [35].

## 2. Materials and Methods

### 2.1. Synthesis of TNTs

Titanate nanotubes were prepared by the alkaline hydrothermal method [21]. In this study, TiO_2_ pigments supplied by Police Chemical Factory (Police, Poland), Tytanpol A11 was used as starting material. TiO_2_-A11, like TiO_2_-P25 produced by Degussa AG (Essen, Germany) is a commercially available photocatalyst. Both photocatalysts are mixtures of two phases, anatase and rutile, but the content of rutile is around 1.6% for TiO_2_-A11 and 18.3% for TiO_2_-P25 [52,53]. 200 mg of the TiO_2_-A11 nanopowder was added to 35 mL of 10 M NaOH (aq.) and sonicated for 30 min. Subsequently, the suspension was transferred to Teflon-lined autoclave and heated at 150 °C for 24 h. The obtained product was sonicated for 30 min and washed with distilled water to lower pH. In the next step, 0.1 M HCl (aq) was gradually added to the system until pH reached 4 and the suspension was washed with distilled water. Obtained TNTs was dried in temperature 55 °C. The resulting powder represents the sample which in this study is denoted as S1.

### 2.2. Modified TNTs

Synthesis of TNTs was modified by adding ultra-small magnetite nanoparticles Fe_3_O_4_ at the stage of washing procedure. Magnetic nanoparticles with an average diameter of about 2 nm were prepared by coprecipitation of Fe^2+^ and Fe^3+^ chlorides (in molar ratio 1:2) in ammonium solution directly in the pores of mesoporous silica MCM-41 (Sigma-Aldrich Sp. z o.o., Poznan, Poland). The details of this method and characteristic of the magnetic material are presented in the previous work [54]. It is important to mention that the use of this method of synthesis of ultra-small magnetic nanoparticles Fe_3_O_4_ makes it possible to appear of a small fraction of γ-Fe_2_O_3_ nanoparticles [54]. The magnetic nanoparticles were extracted from silica by dissolving it in 4 M NaOH (aq.). The solution was sonicated for 60 min and next centrifuged. The supernatant including 2.4 mg of Fe_3_O_4_ nanoparticles (determined with the thiocyanate method) was added during the washing procedure, after the hydrothermal process. All next steps were repeated as in the case of titanate nanotubes synthesis. The resulting powder represents the sample denoted as S2.

### 2.3. Photocatalytic Examination

The photocatalytic activity of TNTs was tested with methylene blue (MB), which is a commonly used substance for that kind of process [55,56]. First, 10 mg of sample (S1, S2) was dispersed in 130 mL of 20 mg/L MB water solution with a pH value of 7 at room temperature. Subsequently, the suspension was mixed (600 rpm) for 60 min in the dark and irradiated with UV light using a high-pressure mercury lamp (Philips, Amsterdam, Holand, 150 W). During the stirring, at various periods, approximately 2 mL of suspension was taken out and centrifuged (14,000 rpm). The absorbance of the supernatant was measured using a UV-Vis spectrophotometer at a certain wavelength λ=664 nm.

### 2.4. Measuring Techniques

Titanate nanotubes were investigated with the help of the following measurement techniques:

- transmission electron microscopy (TEM) by using the Fei Tecnai G2 F20 S Twin transmission electron microscope (Hillsboro, OR, USA) was used,
- EPR measurements by using the BRUKER E500 EPR spectrometer (Billerica, MA, USA) working at 9.4 GHz (X-band) with 100 kHz magnetic field modulation,
- XRD measurements were performed using a Bruker D8 Advance (Billerica, MA, USA) with Johansson monochromator ($\lambda_{\mathrm{Cu}K_{\mathrm{ff}1}}=1.5406$ Å) and detector LynxEye.
- dc magnetization was measured using a Quantum Design Magnetic Property Measurements System MPMS XL-7 (Quantum Design Inc, San Diego, CA, USA) with a superconducting quantum interference device magnetometer (SQUID).

## 3. Results and Discussion

The following subsections present the effect of Fe^3+^-doping on titanate nanotubes using several measurement methods, TEM/EDS microscopy, electron paramagnetic resonance (EPR), Zero Field Cooled/Field Cooled (ZFC/FC) measurements and the photocatalytic properties of modified nanotubes.

### 3.1. TEM Analysis

**Figure 1:**
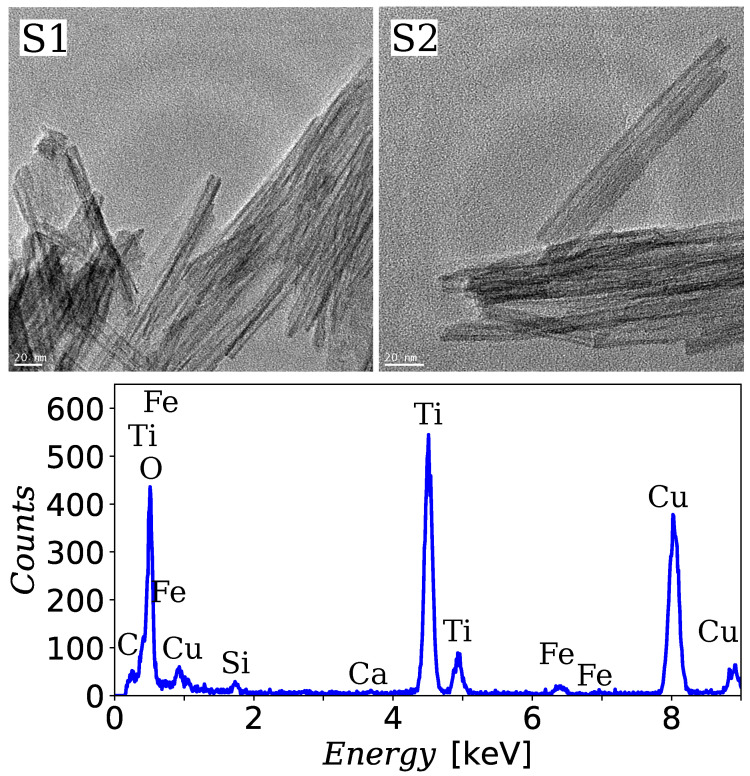
The upper two panels show representative transmission electron microscopy (TEM) images of samples S1 and S2. The lower panel shows the EDS spectrum for the S2 sample with characteristic peaks indicating the presence of iron ions.

TNT product resulting from the hydrothermally changed TiO_2_ nanoparticles is represented by an open-ended multiwall tubelike structure with the spiral cross-section. In this study, the outer and inner diameter of the synthesized nanotubes is about 10 nm and 4 nm, respectively. The length of the nanotube can reach about several hundred nanometers. In Figure 1, the representative TEM images of the prepared titanate nanotubes are shown in the case of the sample S1 and S2. Besides, the results of the elemental analysis of sample S2 provided by the TEM microscopy is presented on the bottom panel. TEM images do not show any difference between nanotubes from samples S1 and S2. In particular, the presence of magnetic nanoparticles cannot be seen in the image of the S2 sample. This may suggest that magnetic nanoparticles that were added to the TiO_2_ material at the stage of the washing procedure simply were absorbed into the structure of the forming nanotubes or they were dissolved in the washing solution. However, the thiocyanate method did not show iron in the supernatant after centrifugation.

### 3.2. Powder XRD Analysis

**Figure 2:**
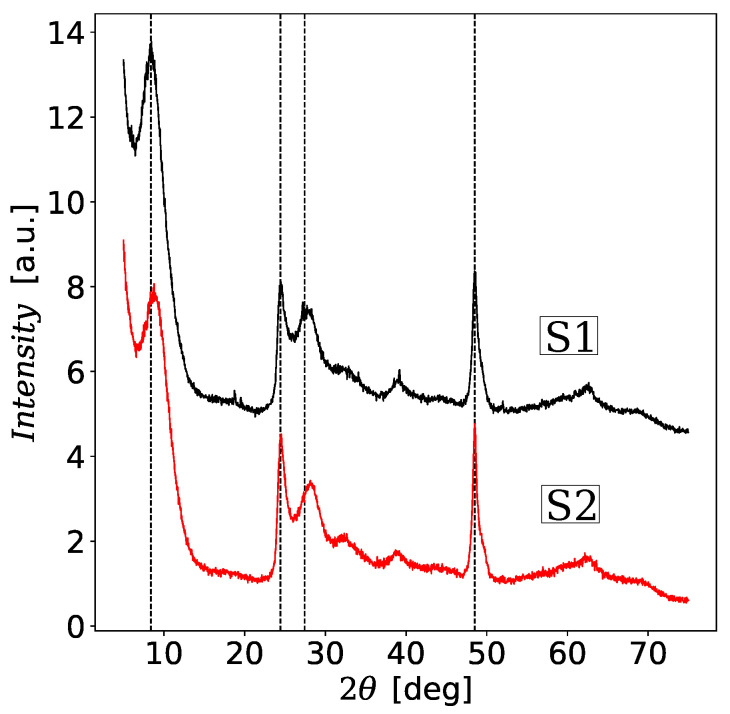
Wide-angle XRD patterns of unmodified titanate nanotubes (TNTs) (sample S1) and modified TNTs (sample S2). The plots have been shifted relative to each other for greater clarity. Dashed vertical lines indicate slight differences in the peaks between S1 and S2 samples such as their location, shape or height.

**Figure 3:**
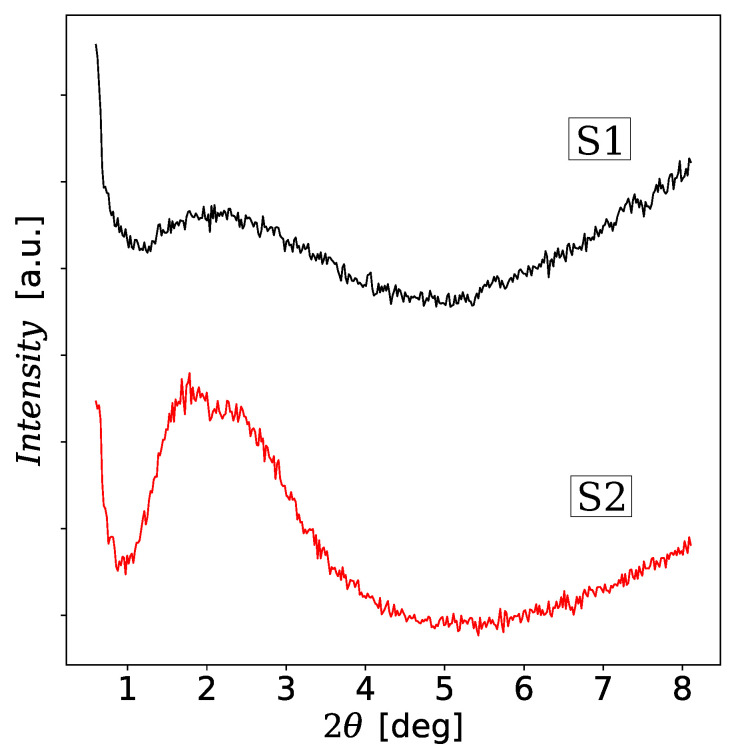
Low-angle XRD patterns of unmodified TNTs (sample S1) and modified TNTs (sample S2). The plots have been shifted relative to each other for greater clarity.

Samples S1 and S2 were also analyzed using the wide-range and low-range X-ray diffraction methods (XRD) which are presented in Figure 2 and Figure 3. The XRD patterns in Figure 2 contain characteristic broadened peaks that resemble the XRD pattern of the trititanic acid (H_2_Ti_3_O_7_). The diffraction pattern of S1 is consistent with the studies by Chen et al. [57] for unmodified TNTs. Some differences can be seen between the diffraction spectra for S1 and S2. The largest differences are marked with vertical lines. In particular, the diffraction peak near $2\theta =10^{{}^{\circ}}$ which represents the interlayer distance of TNT is shifted from $2\theta =8.48^{{}^{\circ}}$ for S1 to 8.79° for S2, i.e., the interlayer distance in the modified TNT decreased. This result is opposite to the observation of the increase in the distance between the interlayer layers of Fe-TNT prepared by the method published in paper [37]. However, in the paper [37] no hydrochloric acid solution was used during the washing procedure, which is crucial for the degradation of iron oxide nanoparticles, which become a source of embedded iron ions. Consequently, we also did not observe additional peaks at 2θ near 33.6°, 35.6°, 40.0°, 44.7°, 62.5° and 64.0° evident in XRD pattern for Fe-TNTs which were assigned to *α*-Fe_2_O_3_ phase [37]. It should be noted that the low-range XRD patterns in Figure 3 also do not show the evidence for loading magnetic nanoparticles into the interior pore structure of TNT. The intensity of the main diffraction peak increased instead. This could mean that there are fewer defects, e.g., in the form of not fully formed nanotubes. All this suggests that the magnetic nanoparticles have degraded during the washing stage of the hydrothermal synthesis giving a contribution to Fe^3+^ substitutional ions doping TNT.

### 3.3. EPR Analysis

**Figure 4:**
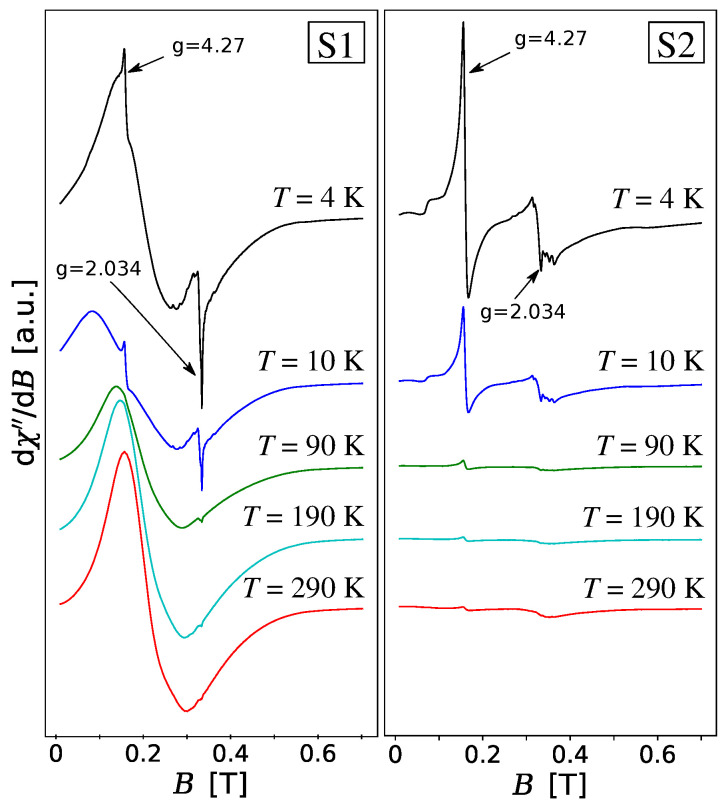
Absorption lines derivatives, d$\chi^{\prime \prime }$/d*B*, of the samples S1 and S2 at different temperatures. The plots have been shifted relative to each other for greater clarity.

The electron paramagnetic resonance (EPR) investigations on TiO_2_ based materials have been carried out for many years [58,59,60]. EPR is a spectroscopic method used to detect unpaired electrons in materials. Commonly studied defects in TiO_2_ by this method are oxygen vacancies, interstitial titanium ions and impurities. The most common impurities in TiO_2_ are Fe^3+^, Cr^3+^, Cu^2+^ and Mn^2+^. In Figure 4, the EPR spectra for samples S1 and S2 have been shown in a wide temperature range from 4 K to 290 K. At low temperatures, two main EPR signals from isolated defects with Fe^3+^ ions and oxygen vacancies are very clearly visible. The intense signal on the left with *g*-factor equal to 4.27 (resonance field B≈158 mT) suggests the existence of Fe^3+^ substitution ions for Ti^4+^ whereas the EPR signal with g=2.034 (resonance field B≈332 mT) can be due to Fe^3+^ ions coupled by exchange interaction with oxygen vacancies. For both samples, the location of the signal Fe^3+^ does not depend on temperature, but its intensity does. At low temperatures, Cu^+2^ impurities are well visible on both samples S1 and S2. Their EPR signals have a characteristic hyperfine structure which does not affect the main peaks and which provides *g*-factor values of approximately 2.5, 2.4 2.3, 2.1 with A≈15 mT. The additional six signals with A≈9 mT around the line with g=2.034 are also evident at low temperatures on both samples S1 and S2. They result from the oxygen vacancies with trapped electrons and they may originate from the other electron trapping sites. The analogous six signals around the line with g=2.034 were observed in [61] for TiO_2_ and Fe-TiO_2_-delaminated clays. It was suggested that they result from the hyperfine interactions between trapped electrons and titanium nuclei. The question arises whether these six signals can be caused by Mn^2+^ impurities which can often be found in TiO_2_ materials. However, in this case, A≈11 mT instead of the observed value of about 9 mT. Example of research with the use of EPR analysis of hydrothermally synthesized Mn^2+^-doped titanate nanotubes can be found in paper [42].

**Figure 5:**
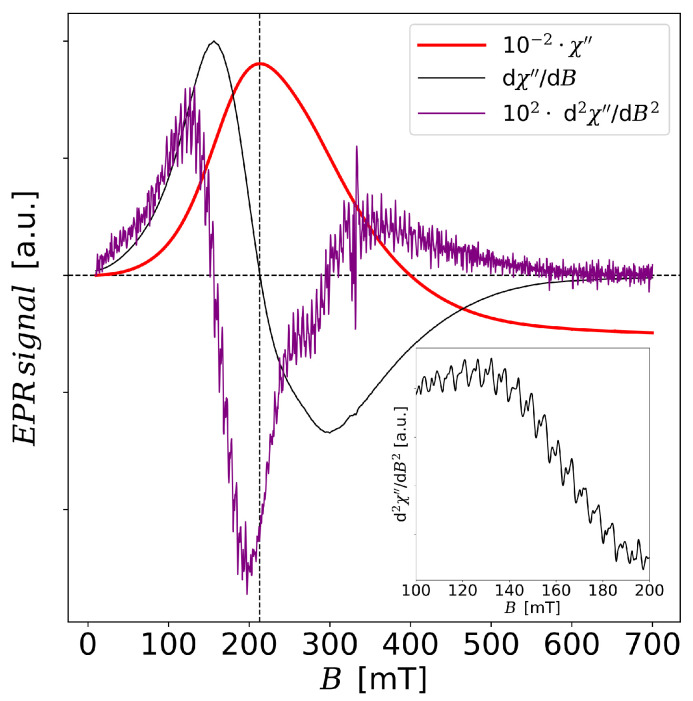
EPR signals (absorption line, first and second order derivative of the absorption line) of sample S1 at temperature T=290 K. The plots have been rescaled for greater clarity. The inset shows a fragment of the second derivative of the absorption line.

The shape of EPR spectral lines measured for samples S1 and S2 changes with temperature but they are qualitatively different. The absorption line derivatives measured for sample S1 at higher temperatures become similar to the spectrum typical for a system with ferromagnetic interactions. It can be observed that the absorption peak ($\chi^{\prime \prime }$) is transforming into one broad peak with a maximum in B≈213 mT where *g* ≈3.17. It represents a multimodal signal as seen in Figure 5 using the second derivative from the absorption line $d^{2}\chi^{\prime \prime }$/d$B^{2}$. These peaks are located centrally on two main lines with g=4.27 and g=2.034 in a form of two series of peaks where the distance between the neighboring peaks is equal to about 6 mT. The presence of these equidistant signals suggests the appearance of structural defects in nanotubes. Probably they arise during the washing process in the hydrothermal method when titanate flakes undergo conversion into the multilayer nanotubes with a spirallike crossection. The nanotubes in sample S1 show a room temperature ferromagnetism which can be observed also for the values of the magnetic field *B* in the region of the main EPR resonances (see Section 3.4). In consequence, the additional demagnetization field $B_{d}$ should be included in the magnetic resonance condition, i.e., $h\nu =g\mu_{B}\left(B+B_{d}\right)$. In the particular case of a long cylinder and magnetic field *B* oriented in the *z*-direction $B_{d}=2\pi M_{z}$ where $M_{z}$ denotes sample magnetization in *z*-direction. The presence of the equidistant EPR signals would mean the presence of the fractions of the correlated spins giving a collective contribution to nanotube magnetization. We should note that sample S2 also shows the ferromagnetic behavior at room temperature. However, for the values of *B* near EPR resonances, the sample is paramagnetic (see Section 3.4). The resulting EPR signal is noisy but it does not show the periodicity observed in S1. Two main resonance lines with g=4.27 and g=2.034 are well distinguished also at higher temperatures.

### 3.4. Magnetic Properties

**Figure 6:**
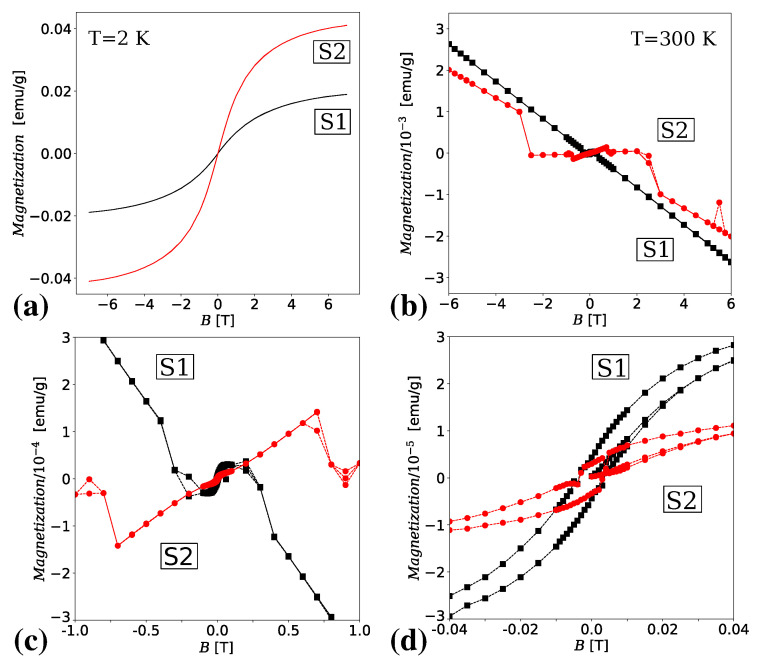
Magnetization vs. dc magnetic field *B* for samples S1 and S2 at temperature T=2 K (**a**), and T=300 K (**b**). Panels (**c**,**d**) show the enlarged fragments of the plots in panel (**b**) for *B* in the range of −1 to 1 T and −0.04 T to 0.04 T, respectively.

Magnetic properties of S1 and S2 samples at temperatures T=2 K and T=300 K are shown in Figure 6 through the plots of their magnetization as a function of the external dc magnetic field *B*. These plots suggest paramagnetic properties at low temperatures (no hysteresis loop) for both samples. If we compare magnetization in sample S2 with the magnetization of 2 nm magnetic nanoparticles Fe_3_O_4_ in mesoporous silica (see Figure 6 in paper [54]), it can be seen that the magnetization values per gram for S2 are smaller by two orders of magnitude in the examined magnetic field range. This is one more confirmation explaining the lack of magnetic nanoparticles in TEM images for S2. However, the magnetization of TNTs in S2 is about twice as high at T=2 K compared to S1.

**Figure 7:**
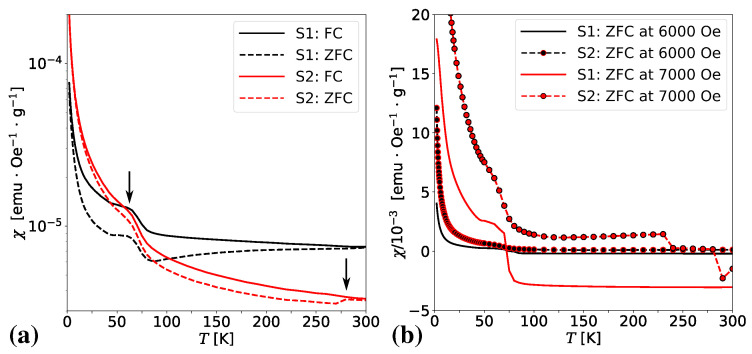
Temperature dependence of Zero Field Cooled/Field Cooled (ZFC/FC) magnetic susceptibility at 100 Oe for samples S1 and S2 (**a**), and temperature dependence of ZFC magnetic susceptibility at large values of the magnetic field, B=6000 Oe and 7000 Oe (**b**). The arrows in (**a**) suggest the change of the type of magnetic response to the applied magnetic field.

It is evident from panel (b) in Figure 6 that the magnetic properties of S1 and S2 at room temperature are much more complex. Both samples show diamagnetic response to the high value of the external magnetic field. However, they show different behavior for low field values. In the case of S1 the diamagnetic response to the applied magnetic field is changed to the ferromagnetic one for a weak magnetic field with the values of *B* ranging from −300 mT to 300 mT. However, there can be observed two different ferromagnetic responses for the values of *B* from −100 mT to 100 mT and beyond these values. The ferromagnetic behavior can be observed also in the case of sample S2 but it is preceded by two different paramagnetic responses to the applied magnetic field with the values in the regions of B≈±0.5 T and B≈±2 T. The magnified fragment of the hysteresis loop diagram for S2 in panel (d) shows discontinuity. Note its absence in sample S1. The discontinuous magnetic response to the applied magnetic field is also suggested in Figure 7 where semi-logarithmic plots of zero-field-cooling (ZFC) and field-cooling (FC) magnetic susceptibilities were shown for samples S1 and S2. The semi-logarithmic scale was used to simultaneously show details of the ZFC/FC relationship for samples S1 and S2. Right arrow around T≈270 K suggests a first-order phase transition. Its origin can be the antiferromagnetic superexchange interaction between Fe^3+^ ions by analogy to the ordering mechanism in hematite (*α*-Fe_2_O_3_) [62].

Panel (a) in Figure 7 shows the temperature dependence of ZFC and FC magnetic susceptibility plots at a magnetic field equal to 0.01 T (100 Oe) with the evident splitting between ZFC and FC curves. For both samples S1 and S2, qualitatively the same behavior below the temperature of about 70 K (pointed by the left arrow) can be observed, i.e., a sharp increase in the magnetic susceptibility of both ZFC and FC. This property usually applies to paramagnetic systems and is a surface effect. In the case of samples S1 and S2, it is the surface effect on the non-interacting magnetic moments suggesting the presence of a paramagnetic phase. Applying stronger magnetic fields to the system causes the ZFC and FC curves to overlap. Panel (b) in Figure 7 shows the dependence of ZFC/ FC magnetic susceptibility on temperature for large magnetic field values equal to 0.6 T and 0.7 T, respectively. The temperature T≈70 K becomes a transition temperature between a paramagnetic response to the applied magnetic field and diamagnetic one of sample S1.

Since the discovery of room temperature ferromagnetism in Co-doped TiO_2_ thin films [63] understanding the causes of ferromagnetism in TiO_2_ materials is a subject of lively discussion [64,65,66,67,68,69]. The hysteresis loop diagram for S1 resembles the results obtained in paper [70] for TiO_2_ single crystals annealed in a high vacuum where the investigated samples were diamagnetic at room temperature with a characteristic low-field ferromagnetic behavior. In paper [70], it was concluded that the unpaired 3d electrons in Ti^3+^ ions can be responsible for the observed room temperature ferromagnetism. In sample S1, a similar mechanism can explain its ferromagnetic properties at a weak magnetic field. The appearance of the equidistant signals on EPR spectral lines with *g*-factors equal to 4.27 and 2.034 can suggest the presence of defects caused by the nanotubular form of TNT.

The magnetic properties of ion-doped titanate nanotubes strongly depend on the method of their preparation and the type of ions used. For example, Fe^3+^-doping method presented in [34] yields hysteresis loops suggesting both paramagnetic and ferromagnetic contributions, the Co-doped titanate nanotubes in [41] show room temperature ferromagnetism, Gd^3+^-doped titanate nanotubes in [43] show low-temperature paramagnetism, Mn^2+^-doped titanate nanotubes in [42] show room temperature ferromagnetism.

The modified titanate nanotubes exhibiting room temperature ferromagnetism, cause interest in the applications of them in spintronics, e.g., Co-doped nanotubes [41]. Another potential field to be used in applications is the exploitation of the magnetocaloric potential of the modified nanotubes. The particular example can be the Gd^3+^-doped titanate nanotubes which were suggested in [43] to be used as a magnetic refrigerant in the temperature range from 5 to 100 K. In the case of S2 nanotubes, their magnetic properties at room temperature are more promising for potential magnetocaloric applications. This may be suggested by the results of the magnetization measurements in Figure 6 which show that a small value of the applied field can change the type of magnetic response from paramagnetic to ferromagnetic and vice versa.

### 3.5. Photocatalytic Effect

**Figure 8:**
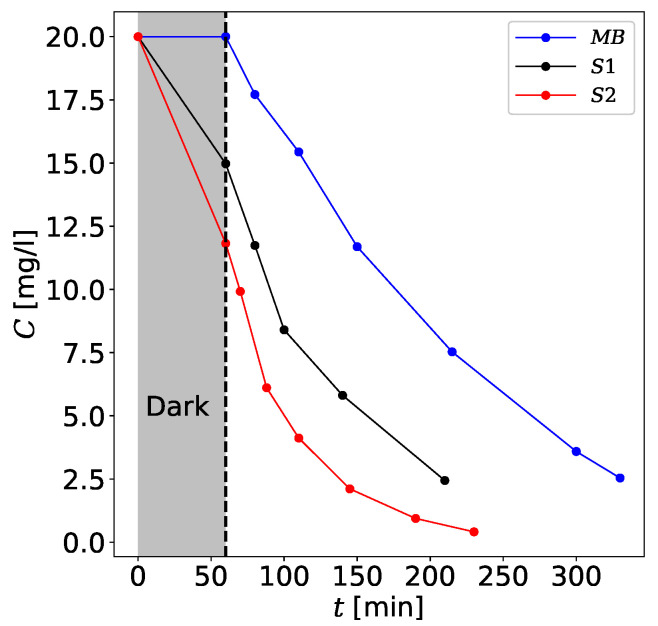
MB concentration *C* as a function of during irradiation with a UV lamp. The plots represent degradation of MB with the photocatalysts S1 and S2, and without them. “Dark” denotes the period of time when the UV lamp was turned off.

Quality of photocatalytic properties can be another test to differentiate samples S1 and S2. For this purpose, the results of the experiment with the degradation of methylene blue (MB) are discussed in the presence of photocatalysts S1 and S2 and without them. They are presented in Figure 8 in terms of the dependence of MB concentration on time in the dark and in the case of irradiation with a UV lamp. Three plots represent the degradation of MB without photocatalysts (effect of photolysis [71]), degradation of MB in the presence of sample S1, and the presence of sample S2, respectively.

The kinetics of photocatalytic decomposition of organic dyes can by successfully represented with the Langmuir-Hinshelwood model [72]. In the case of dilute solutions:

(1) $$\ln\left(\frac{C_{0}}{C}\right)=k_{app}t,$$

where $k_{app}$ is the apparent rate constant of a reaction of pseudo-first-order, $C_{0}$ is the initial pollutant concentration (mg/L) and *C* is the concentration (mg/L) of pollutant at reaction time *t*.

Fe^3+^-doped nanotubes in sample S1 have greater photocatalytic degradability compared to unmodified TNTs in sample S2 [see Table 1]. One of the reasons is moving the edge of light absorption towards the visible light region. The density functional (DFT) calculations performed by Wang et al. [73] showed that transition metal atoms with unpaired electrons in the 3d orbital or 4d orbital contribute to narrowing the bandgap and providing an intermediate energy level. In our case, the light source is a high-pressure mercury lamp and its spectrum contains visible light. Moreover, the Fe^3+^ doping can promote the trapping of the charge carriers. Mahmoud et al. [74] showed that the presence of Fe^3+^ in the titanate nanotube acts as an electron-acceptor and hole-donor. Hence, the lifetime of the electron-hole separation in titanate material increases significantly. In some cases, the presence of Fe^3+^ ions in the structure of titanate nanotubes can drastically lower photocatalytic activity. Jang et al. [75] showed a lower rate of both hydrogen evolution and dye decomposition of Fe-intercalated TNTs than unmodified TNTs. It is worth to mention that the process of TNTs modification, which was used by them, was conducted during hydrothermal synthesis. However, the amount of iron was an order greater than in the present study. It should be noted that the efficiency of photocatalytic processes strongly depends on the concentration of Fe^3+^ ions. Yu et al. [76] noticed that an optimal Fe concentration in Fe-TiO_2_ nanorods is 0.5 atomic%.

## 4. Conclusions

Various forms of TiO_2_-based semiconductor materials such as crystal forms, nanoparticles, flakes or their nanotube forms have been known and studied for many years. These materials are easy to synthesize but the product strongly depends on its preparation. It is the reason for observing different magnetic properties of TNTs for different preparation methods. The results of introducing iron substitutional defects into TNTs show both the presence of the paramagnetic properties of TNTs at low temperatures but also a number of different magnetic responses for different values of the applied magnetic field at room temperature. The room temperature magnetic properties are the most promising for potential applications because the value of the magnetic field driving the magnetization to change from paramagnetic to ferromagnetic behavior and vice versa is relatively small (∼0.07 T). The obtained material with Fe-doped titanate nanotubes also shows increased photocatalytic properties compared to unmodified nanotubes.

## Figures and Tables

**Table 1:** Rate constants $k_{app}$ of photocatalytic methylene blue (MB) degradation and linear regression corelation coefficients $R^{2}$.

Sample	$k_{app}\times 10^{-3}$ [$\min^{-1}$]	$R^{2}$
MB	7.549	0.990
S1	11.933	0.996
S2	19.549	0.998

## Abbreviations

The following abbreviations are used in this manuscript:

EDS	Energy Dispersive X-ray spectroscopy
MB	Methylene Blue
MCM-41	Mobil Composition of Matter No. 41
DFT	Density Functional Theory
EPR	Electron Paramagnetic Resonance
TEM	Transmission Electron Microscopy
TNT	Titanate Nanotubes
XRD	X-ray Powder Diffraction
UV-Vis	Ultraviolet - Visible
ZFC/FC	Zero Field Cooled/Field Cooled
